# Supplementary material for: AI-first structural identification of pathogenic protein target interfaces
Source: PLoS Comput Biol. 2025 Jun 26;21(6):e1013168. doi: 10.1371/journal.pcbi.1013168 (PMC12225977; doi:10.1371/journal.pcbi.1013168)
Supplement: S1 Appendix — Fig A. a) Comparison of pDockQ and the TM-score using FoldDock (n = 111) and FoldDock+templates. The points represent each individual model, the solid lines the running averages using a step size of 0.1 in pDockQ and the dashed grey line a cutoff of 0.3 in pDockQ. When the pDockQ score is high, so is the TM-score. There are more models with low TM-scores at high pDockQ scores using FoldDock+templates. b) ROC curve using pDockQ as a separator for the 111 HP-PPIs with known structure for the standard FoldDock approach (std) and using FoldDock+templates (templates). Positive examples here have a TM-score over 0.9. At an FPR of 5%, 87% of the TP models can be called correct using the std FoldDock model. Fig B. An example of an accurate prediction (6OAM_A-6OAM_d), where DockQ reports a low score (DockQ = 0.007) and MMalign a high score (TM-score = 0.97). This exemplifies the need to take the length difference between native and predicted structures into account and supports the use of MMalign. Fig C. Example of an interaction where one chain is predicted to intersect the other, although there are no clashes (Q14318-Q8D097). These cases were removed as well. Fig D. Analysis of the high quality HP-PPIs for HPV. The human proteins are shown in green and the pathogenic ones in cyan. Potential native structures are shown in grey superposed with the predictions. a) Predicted structure of the interaction between UBA1 (https://www.uniprot.org/uniprot/P22314) and E2 (https://www.uniprot.org/uniprot/P03120) with the interface residues coloured in magenta. UBA1 is in structural superposition with the native structure (grey, TM-score = 0.98). The native structure of UBA1 is complex with ubiquitin (PDB ID 6DC6, https://www.rcsb.org/structure/6dc6) and with E2 superposed according to the predicted structure. E2 captures ubiquitin in its activation area and thereby likely prevents its release. b) Interaction between SRP19 (https://www.uniprot.org/uniprot/P09132), crucial for ribosome b [file pcbi.1013168.s001.pdf]

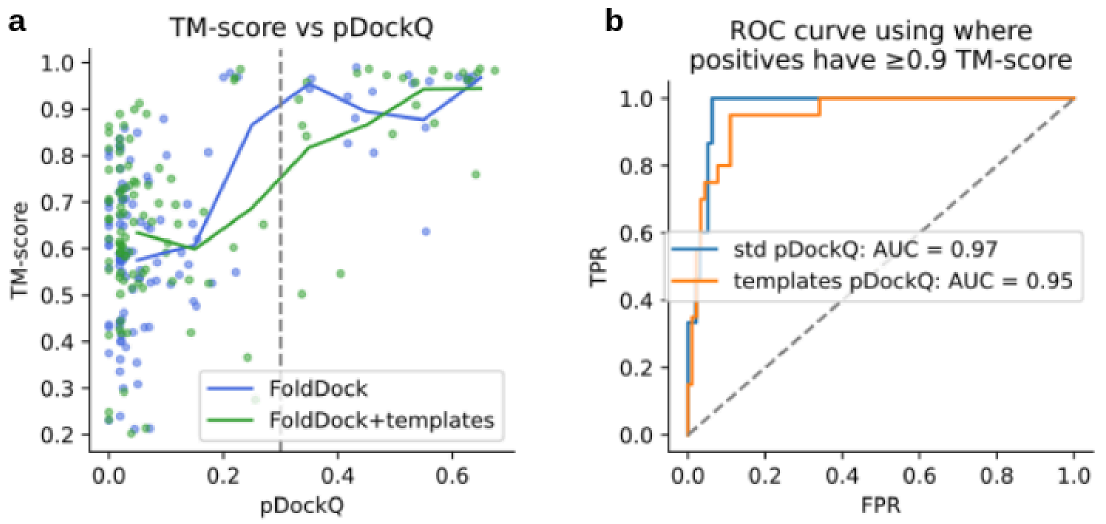

Fig A. a) Comparison of pDockQ and the TM-score using FoldDock (n=111) and FoldDock+templates. The points represent each individual model, the solid lines the running averages using a step size of 0.1 in pDockQ and the dashed grey line a cutoff of 0.3 in pDockQ. When the pDockQ score is high, so is the TM-score. There are more models with low TM-scores at high pDockQ scores using FoldDock+templates. **b)** ROC curve using pDockQ as a separator for the 111 HP-PPIs with known structure for the standard FoldDock approach (std) and using FoldDock+templates (templates). Positive examples here have a TM-score over 0.9. At an FPR of 5%, 87% of the TP models can be called correct using the std FoldDock model.

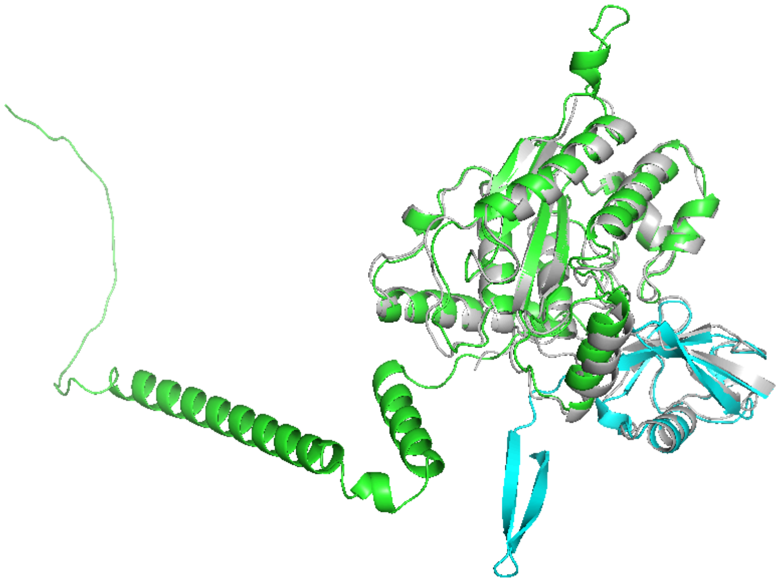

Fig B. An example of an accurate prediction (6OAM_A-6OAM_d), where DockQ reports a low score **(**DockQ = 0.007) and MMalign a high score (TM-score = 0.97). This exemplifies

the need to take the length difference between native and predicted structures into account and supports the use of MMalign.

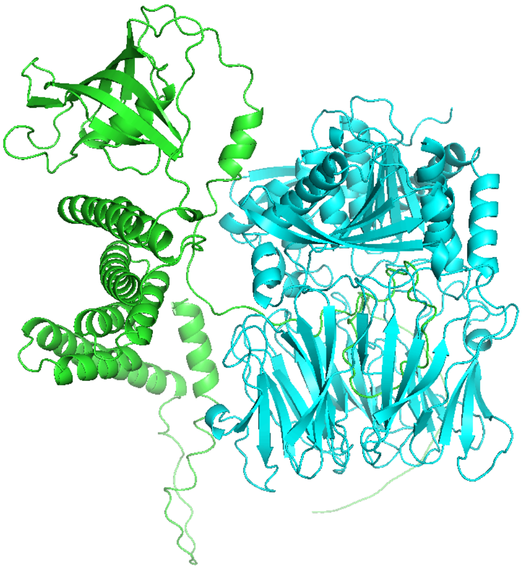

Fig C. Example of an interaction where one chain is predicted to intersect the other, although there are no clashes (Q14318-Q8D097). These cases were removed as well.

Table A. The number of structures in the PDB, the number of HP-PPIs in the HPIDB, the number of successful predictions, the number of these where both proteins have an average pIDDT>70 (single chain, sc, pIDDT) and high-confidence predictions (sc pIDDT>70 and pDockQ>0.3) obtained here for human-pathogen protein-protein interactions divided by pathogen.

Pathogen	Structures in PDB	Number of HP-PPIs in the HPIDB	Number of successful predictions	Number of predictions with sc pIDDT>70	High confidence predictions (sc pIDDT>70, pDockQ>0.3)	Total
Bacillus anthracis	2	2882	2471	1170	8	10
Epstein-Barr virus	4	244	238	37	1	5
Human papillomavirus type 16	3	171	158	59	5	8
Influenza A virus	1	238	230	53	0	1
Francisella tularensis subsp.	0	1295	1146	518	4	4

tularensis						
Hepatitis C virus genotype 1b	0	442	389	91	0	0
Yersinia pestis	0	3856	3405	1586	25	25
Dengue virus type 2	0	134	104	30	0	0
Human T-cell leukemia virus 1	0	77	76	0	0	0
Coxiella burnetii	0	237	224	49	0	0
Total	10	9576	8441	3593	43	53

High-quality predictions from the HPIDB

All structures displayed in this section are predictions using the FoldDock [1] pipeline based on AlphaFold2 [2]. We analyse the predicted interactions in the context of previous findings to suggest potential induced mechanisms.

Human papillomavirus type 16

Fig Da shows the target selected for MS testing (see the main text). Fig Db shows the interaction between SRP19 (https://www.uniprot.org/uniprot/P09132), crucial for ribosome binding, and HPV protein E7 (https://www.uniprot.org/uniprot/P03129), which is involved in regulatory mechanisms such as transcriptional activation. In its native form, SRP19 interacts with RNA (https://www.rcsb.org/structure/1JID). E7 is, however, predicted to occupy the same position as the RNA, suggesting a potential inhibitory mechanism. FigD c-e shows interactions between various forms of human proteins which facilitate nuclear import of other proteins (TNPO1 https://www.uniprot.org/uniprot/Q92973, KPNB1 https://www.uniprot.org/uniprot/Q14974 and IPO5 https://www.uniprot.org/uniprot/O00410) and viral proteins E6 (an oncoprotein that destroys many host cell regulatory proteins, https://www.uniprot.org/uniprot/P03126) and L1 (the viral capsid protein, https://www.uniprot.org/uniprot/P03101).

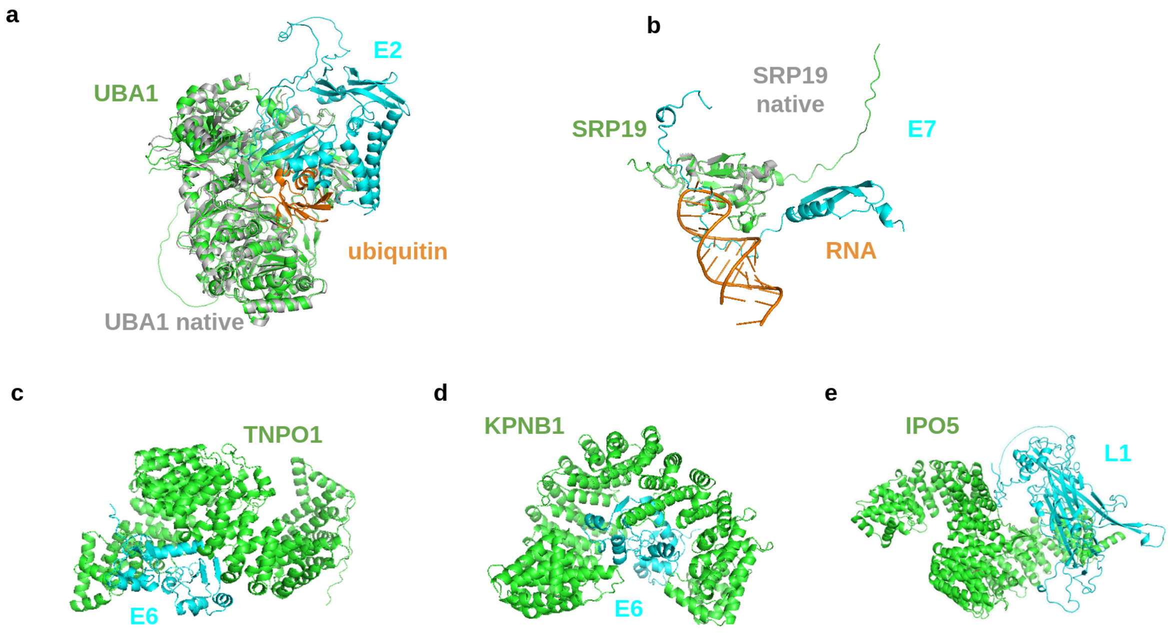

Fig D. Analysis of the high quality HP-PPIs for HPV. The human proteins are shown in green and the pathogenic ones in cyan. Potential native structures are shown in grey superposed with the predictions. a) Predicted structure of the interaction between UBA1 (https://www.uniprot.org/uniprot/P22314) and E2 (https://www.uniprot.org/uniprot/P03120) with the interface residues coloured in magenta. UBA1 is in structural superposition with the native structure (grey, TM-score = 0.98). The native structure of UBA1 is complex with ubiquitin (PDB ID 6DC6, https://www.rcsb.org/structure/6dc6) and with E2 superposed according to the predicted structure. E2 captures ubiquitin in its activation area and thereby likely prevents its release. b) Interaction between SRP19 (https://www.uniprot.org/uniprot/P09132), crucial for ribosome binding, and HPV protein E7 (https://www.uniprot.org/uniprot/P03129) which is involved in regulatory mechanisms such as transcriptional activation. In its native form, SRP19 interacts with RNA (https://www.rcsb.org/structure/1JID) c-e) Interactions between various forms of human proteins which facilitate nuclear import of other proteins (TNPO1 https://www.uniprot.org/uniprot/Q92973, KPNB1 https://www.uniprot.org/uniprot/Q14974 and IPO5 https://www.uniprot.org/uniprot/O00410) and viral proteins E6 (an oncoprotein that destroys many host cell regulatory proteins, https://www.uniprot.org/uniprot/P03126) and L1 (the viral capsid protein, https://www.uniprot.org/uniprot/P03101).

Epstein-Barr virus

The only high-quality prediction for EBC is between TTC12 (https://www.uniprot.org/uniprot/Q9H892) and BBRF2 (https://www.uniprot.org/uniprot/P29882)**,** Fig E. TTC12 is a protein that plays a role in the assembly of motile cilia and is found in the cytoplasm (no available structure in the PDB). BBRF2 is critical for virus egress and is part of the final step in envelope acquisition from the host cytoplasm (closest structure: https://www.rcsb.org/3d-view/6LQN/1). A possible mode of action for viral egress is that TTC12 helps to assemble the capsid and thereby facilitating viral egress through interactions with BBRF2 (it looks like TTC12 grabs hold of BBRF2).

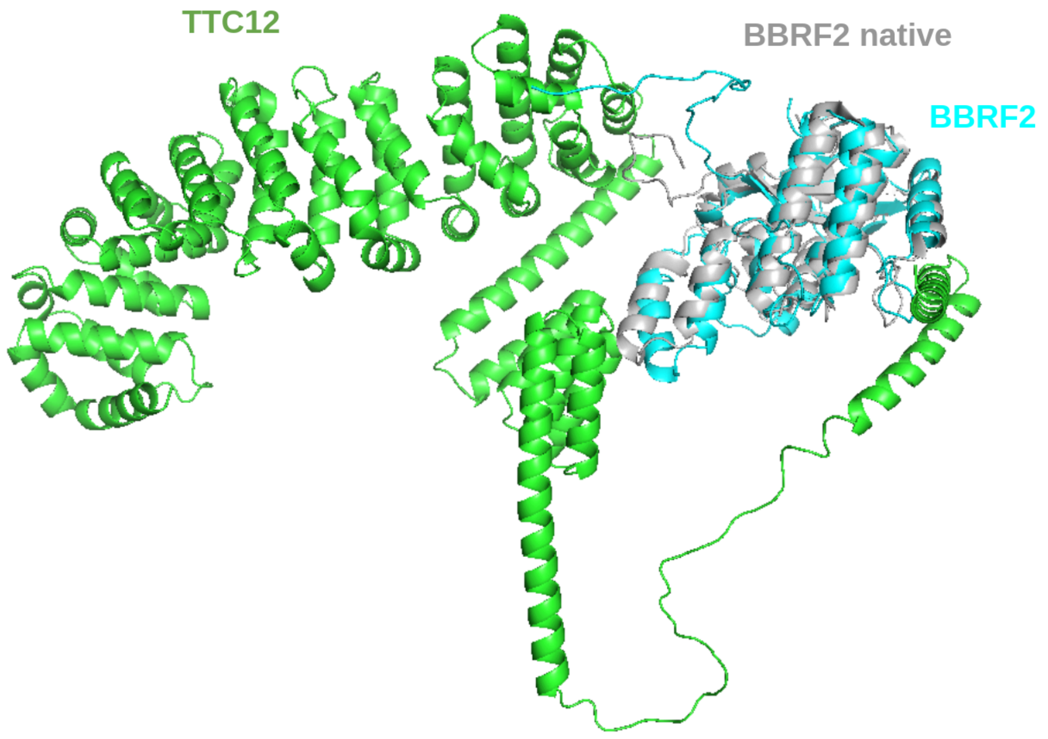

Fig E. Analysis of the high-quality HP-PPI for EBV. The human proteins are shown in green and the pathogenic ones in cyan. Potential native structures are shown in grey superposed with the predictions. Predicted structure of the interaction between TTC12 (https://www.uniprot.org/uniprot/Q9H892) and BBRF2 (https://www.uniprot.org/uniprot/P29882). BBRF2 is in structural superposition with the native structure (grey, https://www.rcsb.org/3d-view/6LQN/1).

Francisella tularensis subsp. *tularensis*

Fig F shows the three high-quality predictions from *F. tularensis*. IGKC (membrane-bound immunoglobulin, https://www.uniprot.org/uniprot/P01834) interacts with IPD (https://www.uniprot.org/uniprot/Q5NEX4) in Fig Fa. It is possible that IPD inhibits the IGKC through this interaction, preventing immune response towards other pathogenic particles. In Fig Fb, TRPC1 (Short transient receptor potential channel 1, https://www.uniprot.org/uniprot/P48995) interacts with purL (Phosphoribosylformylglycinamidine synthase, https://www.uniprot.org/uniprot/Q5NEC0). This interaction seems unlikely as no biological mechanism is supported here. This may be a FP from the experiments due indirect interactions (associative). LNPEP (Leucyl-cystinyl aminopeptidase, https://www.uniprot.org/uniprot/Q9UIQ6) interacts with argS (Arginine-tRNA ligase, https://www.uniprot.org/uniprot/Q5NHI8) in Fig Fc. This interaction may inhibit the activity of LNPEP sterically, although the substrate binding site is not hindered.

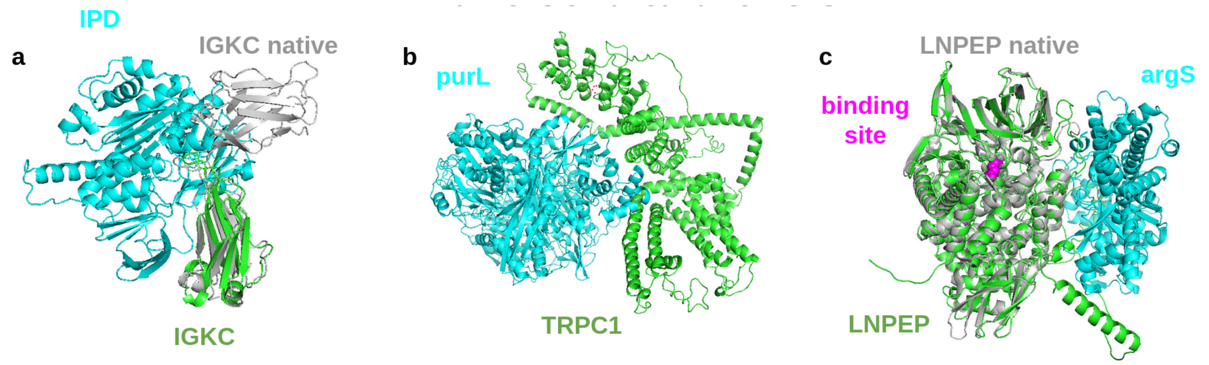

Fig F. Analysis of the high quality HP-PPI for *F. tularensis*. The human proteins are shown in green and the pathogenic ones in cyan. Potential native structures are shown in grey superposed with the predictions. **a)** IGKC (https://www.uniprot.org/uniprot/P01834) interacts with IPD (https://www.uniprot.org/uniprot/Q5NEX4), potentially hindering antibody formation. **b)** TRPC1 (https://www.uniprot.org/uniprot/P48995) interacts with purL (https://www.uniprot.org/uniprot/Q5NEC0). No clear biological mechanism can be deduced from this interaction. **c)** LNPEP (https://www.uniprot.org/uniprot/Q9UIQ6) interacts with argS (https://www.uniprot.org/uniprot/Q5NHI8). This interaction may inhibit the activity of LNPEP sterically, although the substrate binding site (magenta) is not hindered.

Bacillus anthracis

Fig G shows the six high-quality predictions from *Bacillus anthracis*. XPO1 (Exportin-1, https://www.uniprot.org/uniprot/O14980) interacts with dacB (Diadenylate cyclase, https://www.uniprot.org/uniprot/A0A6L8PQ13) in Fig Ga, suggesting nuclear export. PFAS (Phosphoribosylformylglycinamidine synthase, https://www.uniprot.org/uniprot/O15067) interacts with leuS (Leucine-tRNA ligase, https://www.uniprot.org/uniprot/Q81KK6) in Fig Gb, suggesting a potential inhibition of enzyme activity. Fig Gc shows ANXA2 (Annexin A2, https://www.uniprot.org/uniprot/P07355) interacting with GBAA_3695 (Unknown protein, https://www.uniprot.org/uniprot/A0A0F7RE19). This interaction may inhibit the production of reactive oxygen species [3].

In Fig Gd, LMNA (Prelamin A/C, https://www.uniprot.org/uniprot/P02545) interacts with GBAA_0983 (putative membrane protein, https://www.uniprot.org/uniprot/A0A6L8P438). The meaning of this interaction is hard to deduce. CTSB (Cathepsin B, https://www.uniprot.org/uniprot/P07858) interacts with GBAA_0078 (UVR domain-containing protein, https://www.uniprot.org/uniprot/A0A6L8P7D1) in Fig Ge. At the same interaction site, CTSB has been shown to be inhibited by binding to the protein Chagasin from Trypanosoma cruzi [4] preventing protein degradation. The structure of WASHC5 (WASH complex subunit 5, https://www.uniprot.org/uniprot/Q12768) and vpR (Minor extracellular protease VpR, https://www.uniprot.org/uniprot/A0A6H3AKS4), which may alter some type of cellular trafficking.

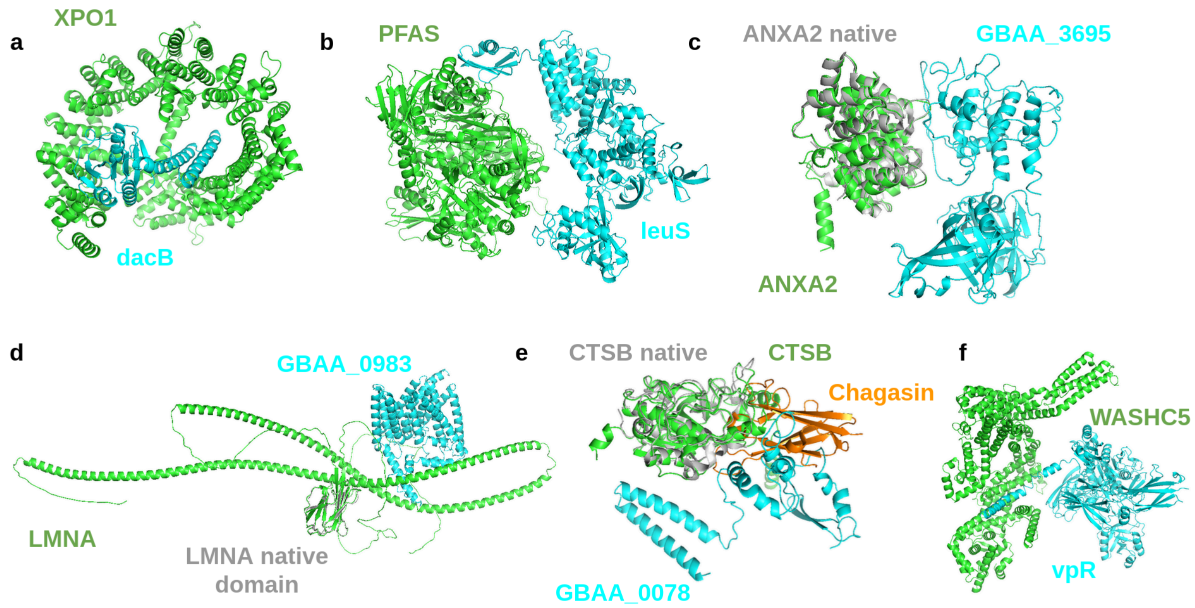

Fig G. Analysis of the high quality HP-PPI for *B. anthracis*. The human proteins are shown in green and the pathogenic ones in cyan. Potential native structures are shown in grey superposed with the predictions. **a)** XPO1 (Exportin-1, https://www.uniprot.org/uniprot/O14980) interacts with dacB (Diadenylate cyclase, https://www.uniprot.org/uniprot/A0A6L8PQ13) **b)** PFAS (Phosphoribosylformylglycinamidine synthase, https://www.uniprot.org/uniprot/O15067) interacts with leuS (Leucine-tRNA ligase, https://www.uniprot.org/uniprot/Q81KK6) **c)** ANXA2 (Annexin A2, https://www.uniprot.org/uniprot/P07355) interacting with GBAA_3695 (Unknown protein, https://www.uniprot.org/uniprot/A0A0F7RE19) **d)** LMNA (Prelamin A/C, https://www.uniprot.org/uniprot/P02545) interacts with GBAA_0983 (putative membrane protein, https://www.uniprot.org/uniprot/A0A6L8P438) **e)** CTSB (Cathepsin B, https://www.uniprot.org/uniprot/P07858) interacts with GBAA_0078 (UVR domain-containing protein, https://www.uniprot.org/uniprot/A0A6L8P7D1). The structure of Chagasin (https://www.rcsb.org/structure/3CBJ) is shown as it is interacting with CTSB. **f)** WASHC5 (WASH complex subunit 5, https://www.uniprot.org/uniprot/Q12768) and vpR (Minor extracellular protease VpR, https://www.uniprot.org/uniprot/A0A6H3AKS4).

Yersinia Pestis

Fig H shows the 14 high-quality predictions from *Yersinia Pestis*. In Fig Ha, PDCD6 (Programmed cell death protein 6, https://www.uniprot.org/uniprot/O75340) interacts with slt (Peptidoglycan lytic exotransglycosylase, https://www.uniprot.org/uniprot/Q8CZP1). HEBP2 is also shown bound to PDCD6 (https://www.rcsb.org/3d-view/5GQQ), which is thought to promote the inhibition of HIV production [5]. It is possible the interaction between slt and PDCD6 works in a similar manner. IGKC (Immunoglobulin kappa constant, https://www.uniprot.org/uniprot/P01834) and yopM (Outer membrane protein, https://www.rcsb.org/structure/1G9U) are interacting in Fig Hb, which may inhibit antibody formation. A similar inhibition is possible in Fig Hc, where IGHA (Immunoglobulin heavy constant alpha 2, https://www.uniprot.org/uniprot/P01877) interacts with tssC (type VI secretion system contractile sheath large subunit, https://www.uniprot.org/uniprot/A0A3N4B420).

The interaction between PRDX3 (Thioredoxin-dependent peroxide reductase, https://www.uniprot.org/uniprot/P30048) and mtfA (Involved in the regulation of ptsG expression by binding and inactivating Mlc, https://www.uniprot.org/uniprot/Q7CHU1) with the oligomeric structure of native PRDX3 (https://www.rcsb.org/structure/5UCX) is displayed in Fig Hd. PRDX3 plays a role in protection against oxidative stress, suggesting that oxidative stress may be promoted by binding to PRDX3. PRDX3 forms a ring structure in its native state, which is why predicting this interaction as dimeric may not be very informative. CRAT (Carnitine O-acetyltransferase, https://www.uniprot.org/uniprot/P43155) and tssC (Fig Hc) interact in Fig He with the CRAT native structure (https://www.rcsb.org/structure/1NM8) displayed in structural superposition. It is possible this interaction inhibits CRAT, preventing oxidation of branched-chain amino acids. B2M (Beta-2-microglobulin, https://www.uniprot.org/uniprot/P61769, superposition with the structure of MHC-I: https://www.rcsb.org/structure/1A1M) interacts with YPMT1.34 (Uncharacterized, https://www.uniprot.org/uniprot/O68752) in Fig Hf, which may have an inhibitory effect on MHC-I formation and thereby antigen presentation.

The interaction between YWHAE (14-3-3 protein epsilon, https://www.uniprot.org/uniprot/P62258) and mukB (Chromosome partition protein MukB, https://www.uniprot.org/uniprot/Q8ZG99) is shown in Fig Hg. YWHAE is an important signalling protein and may be inhibited. Fig Hh shows the interaction between YWHAZ (14-3-3 protein zeta/delta, https://www.uniprot.org/uniprot/P63104) and istA (IS21-like element IS100 family transposase, https://www.uniprot.org/uniprot/Q7ARN5) and may have a similar relationship as in g. CSNK2B(Casein kinase II subunit beta, https://www.uniprot.org/uniprot/P67870) and nifj (Putative pyruvate-flavodoxin oxidoreductase, https://www.uniprot.org/uniprot/A0A3N4BEU0) interact in Fig Hi. CSNK2B participates in Wnt signalling, important for immune surveillance, which means that this may be avoided or down-regulated through the interaction with nifj.

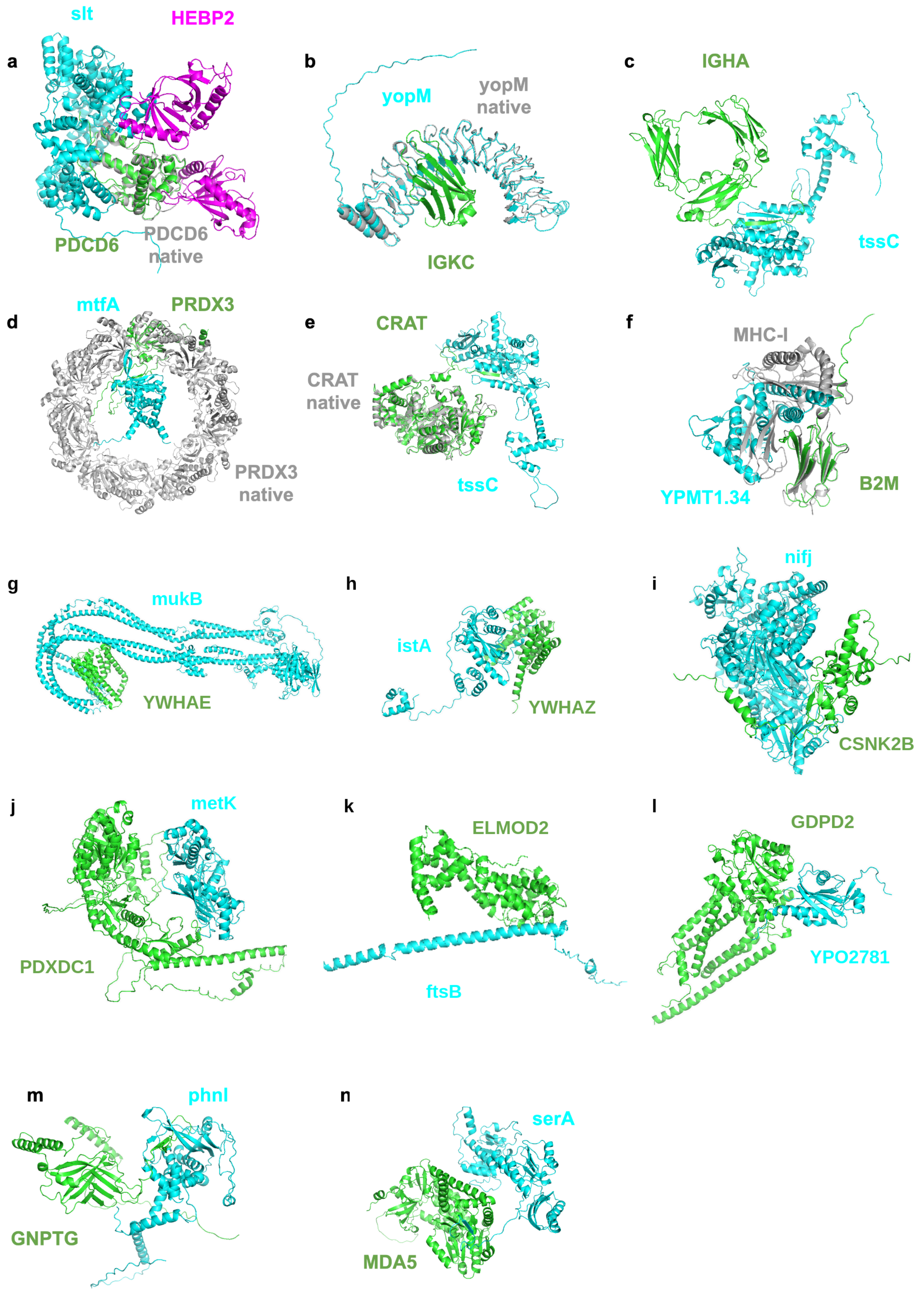

Fig H. Analysis of the high quality HP-PPI for *B. anthracis*. The human proteins are shown in green and the pathogenic ones in cyan. Potential native structures are shown in grey superposed with the predictions.

a) PDCD6 (Programmed cell death protein 6, https://www.uniprot.org/uniprot/O75340) interacts with slt (Peptidoglycan lytic exotransglycosylase, https://www.uniprot.org/uniprot/Q8CZP1). HEBP2 is also shown bound to PDCD6 (https://www.rcsb.org/3d-view/5GQQ), which is thought to promote the inhibition of HIV production. **b)** IGKC (Immunoglobulin kappa constant, https://www.uniprot.org/uniprot/P01834) and yopM (Outer membrane protein, https://www.rcsb.org/structure/1G9U) are interacting, which may inhibit antibody formation. **c)** IGHA (Immunoglobulin heavy constant alpha 2, https://www.uniprot.org/uniprot/P01877) interacts with tssC (type VI secretion system contractile sheath large subunit, https://www.uniprot.org/uniprot/A0A3N4B420). **d)** PRDX3 (Thioredoxin-dependent peroxide reductase, https://www.uniprot.org/uniprot/P30048) and mtfA (Involved in the regulation of ptsG expression by binding and inactivating Mlc, https://www.uniprot.org/uniprot/Q7CHU1) with the oligomeric structure of native PRDX3 (https://www.rcsb.org/structure/5UCX). **e)** CRAT (Carnitine O-acetyltransferase, https://www.uniprot.org/uniprot/P43155) and tssC (S8c Fig) interact with the CRAT native structure (https://www.rcsb.org/structure/1NM8) displayed in structural superposition. **f)** B2M (Beta-2-microglobulin, https://www.uniprot.org/uniprot/P61769, superposition with the structure of MHC-I: https://www.rcsb.org/structure/1A1M) interacts with YPMT1.34 (Uncharacterized, https://www.uniprot.org/uniprot/O68752). **g)** YWHAE (14-3-3 protein epsilon, https://www.uniprot.org/uniprot/P62258) and mukB (Chromosome partition protein MukB, https://www.uniprot.org/uniprot/Q8ZG99). **h)** YWHAZ (14-3-3 protein zeta/delta, https://www.uniprot.org/uniprot/P63104) and istA (IS21-like element IS100 family transposase, https://www.uniprot.org/uniprot/Q7ARN5). **i)** CSNK2B(Casein kinase II subunit beta, https://www.uniprot.org/uniprot/P67870) and nifj (Putative pyruvate-flavodoxin oxidoreductase, https://www.uniprot.org/uniprot/A0A3N4BEU0) interact.

References

1. Bryant P, Pozzati G, Elofsson A. Improved prediction of protein-protein interactions using AlphaFold2. Nat Commun. 2022;13: 1–11.
2. Jumper J, Evans R, Pritzel A, Green T, Figurnov M, Ronneberger O, et al. Highly accurate protein structure prediction with AlphaFold. Nature. 2021;596: 583–589.
3. Alberts A, Klingberg A, Hoffmeister L, Wessig AK, Brand K, Pich A, et al. Binding of Macrophage Receptor MARCO, LDL, and LDLR to Disease-Associated Crystalline Structures. Front Immunol. 2020;0. doi:10.3389/fimmu.2020.596103
4. Redzynia I, Ljunggren A, Abrahamson M, Mort JS, Krupa JC, Jaskolski M, et al. Displacement of the occluding loop by the parasite protein, chagasin, results in efficient inhibition of human cathepsin B. J Biol Chem. 2008;283: 22815–22825.
5. Ma J, Zhang X, Feng Y, Zhang H, Wang X, Zheng Y, et al. Structural and Functional Study of Apoptosis-linked Gene-2·Heme-binding Protein 2 Interactions in HIV-1 Production. J Biol Chem. 2016;291: 26670–26685.
