# Supplementary material for: AI-first structural identification of pathogenic protein target interfaces
Source: PLoS Comput Biol. 2025 Jun 26;21(6):e1013168. doi: 10.1371/journal.pcbi.1013168 (PMC12225977; doi:10.1371/journal.pcbi.1013168)
Supplement: Attachment — Submitted filename: Response to Reviewers.pdf [file pcbi.1013168.s002.pdf]

**We thank the Editor and Reviewers for their insightful comments and have outlined a point-by-point response in bold below. The manuscript changes can be found tracked in red.**

**Reviewers' comments:**

Reviewer's Responses to Questions

**Reviewer #1:** This manuscript utilizes AlphaFold-based protein structure prediction methods to predict the structures of host-pathogen protein-protein interactions (HP-PPIs). The authors applied AlphaFold-Multimer (AFM) and FoldDock protocols to predict the structures of 111 curated HP-PPI complexes, comparing TM-scores to determine that FoldDock is more suitable for HP-PPI prediction. Building upon this, the authors predicted the structures of 9,452 HP-PPIs from the HPIDB database using FoldDock, selecting 30 high-quality predictions based on plDDT ≥ 70 and pDockQ ≥ 0.3 for further analysis. In particular, the authors analyzed the complex between human IGKC and Francisella tularensis IPD and observed that IGKC binds at a distinct site from the IPD dimerization interface. Based on this observation, the authors hypothesized that IPD exists as a dimer and interacts with two IGKC molecules, forming a 1:2:1 heterotetramer structure. To validate this hypothesis, the authors conducted native mass spectrometry (nMS) and confirmed the formation of a 1:2:1 complex, demonstrating that AI-based structure prediction can contribute to identifying biologically relevant protein interactions. This manuscript highlights the potential of AI-driven structural modeling in elucidating host-pathogen protein interactions and predicting novel functional interactions. However, there are several issues to be addressed in the current form of the manuscript.

Major comments

1. The authors selected "high-quality predictions" based on structures with an average plDDT ≥ 70 and pTM-score ≥ 0.9. However, it is questionable whether the average plDDT value and pTM-score across the entire protein structure accurately reflect the reliability of the predicted HP-PPI structures. Since this study aims to predict HP-PPI structures, the plDDT of the binding sites and the PAE values between interacting residues are more relevant. However, this aspect seems to be insufficiently addressed.

**We thank the reviewer for this comment. We use the plDDT of the binding sites only when selecting using pDockQ which is based on the interface plDDT * log(number of contacts) - see the methods section. It is not possible to use PAE with the original AF2 model, as this was added in afterwards. We do believe the original model (and weights) are the best for discovery as these contain the least bias as can be seen when comparing with e.g. AFM (Figure 1b). Even though AFM has been explicitly trained to predict complexes it does not outperform the single-chain AF2 model. The plDDT threshold of 70 was used to account for the bias in PDB which the pDockQ score is calibrated on as explained in the results section but is not used for selecting accurate interfaces:**

*"The structures in PDB will be highly ordered, even considering the full-length sequences. This is because disordered proteins are hard to crystallise and thereby, infrequent in structural databases. In reality, many proteins are highly flexible, and many viruses have highly disordered or non-structural proteins [14]. To account for this bias, we analyse the protein disorder distribution measured by the predicted lDDT (plDDT) from AlphaFold using the HP-PPIs from PDB and compare it to the set from the HPIDB (Figure 2c). As expected, the PDB set has higher plDDT on average. To account for this discrepancy, we select only the complexes where both chains have an average plDDT ≥70"*

2. Along the same line with point 1, the authors mention that they found 30 new structures (p. 4, last paragraph). Do they have any experimental evidence to support that those predictions are reliable?

**We do not have any experimental evidence for these predicted structures and also do not make any such claims. As stated in the abstract: "**We identify 30 interactions with an expected TM-score ≥0.9". **We underline that these structures are, to the best of our knowledge, expected to be accurate according to all analyses, but we do agree that the only way to know would be to solve the complexes. Throughout the manuscript, we don't claim these structures to be true, but predicted, e.g.:**

*"The prediction and identification of high-quality HP-PPI structures enable the extraction of key parts of these complexes. To illustrate the principle of AI-first target identification, all 30* ***high-quality predictions*** *were analysed. "*

3. The authors mention that pathogen proteins tend to be structurally unstable or contain a high proportion of intrinsically disordered regions (IDRs) (Structure prediction of novel host-pathogen interactions section, second paragraph). It is well known that AlphaFold2 exhibits hallucination effects when predicting secondary structures in IDR regions, which may artificially inflate plDDT values and affect PPI predictions. To address this issue, it would be beneficial to compare the predicted structures using more recent AI tools such as AlphaFold3 or Chai.

**IDR regions indeed lead to disordered predictions, but it is AlphaFold3 and not AlphaFold2 that hallucinates and produces ordered structures instead of the "spaghetti" of AF2. DeepMind tried to assess this - albeit unsuccessfully - by fine-tuning AF3 on AF2 predictions (https://www.nature.com/articles/s41586-024-07487-w). The ability of AF2 to predict disorder when unsure allows us to disregard these predictions and focus on the ones we can assess. In addition, it would not be possible to evaluate the ability of tools like AF3 (or copies like Chai-1) to predict host-pathogen interactions as there are not many available in the PDB, and both networks have seen all of these during training, leaving nothing for benchmarking. Therefore, we believe Folddock, based on AF2, is currently the best option for this problem.**

4. Did the authors consider HP-PPI interactions in a physiological environment? In vivo,

these proteins do not exist in a static state. To better assess the stability of the predicted complexes, molecular dynamics (MD) simulations should be performed, demonstrating that the predicted binding site remains stable throughout the simulation. This would strengthen the credibility of the predictions.

**We refer to - and agree with - the editor's comment here.**

5. The authors may consider comparing the predictions of monomeric and complex forms of the same molecule. This can provide additional insight on conformational flexibility, etc.

**We do not understand what "molecule" is referred to here? We have predicted the structure of binary complexes and also do not see how the aspect of conformational flexibility is relevant at this stage as we do not e.g. assess affinity between the individual proteins.**

6. The interpretation of the predicted complexes in Figure 3 appears questionable. For example, in Figure 3i, the authors suggest that CSNK2B is involved in the Wnt signaling pathway and that its interaction with nifj may disrupt Wnt signaling or contribute to immune evasion. However, this claim is purely based on functional inference and lacks structural evidence. To support this hypothesis, it is necessary to confirm and provide evidence that the functional active site of CSNK2B overlaps with the binding site of nifj.

**We thank the reviewer for this comment. One possible form of inhibition is targeting an active site. However, many other forms of inhibition exist, and allosteric inhibition or binding that alters the dynamics of a target is also possible. Therefore, one can't conclude that a target is inhibited or not based on the binding site alone. In the case of [https://www.rcsb.org/structure/1DS5](https://www.rcsb.org/structure/1DS5) it seems like the pathogen interaction may result in inhibition of the interaction with the second subunit of CSNK2B together forming the active site (spheres, cyan/magenta):**

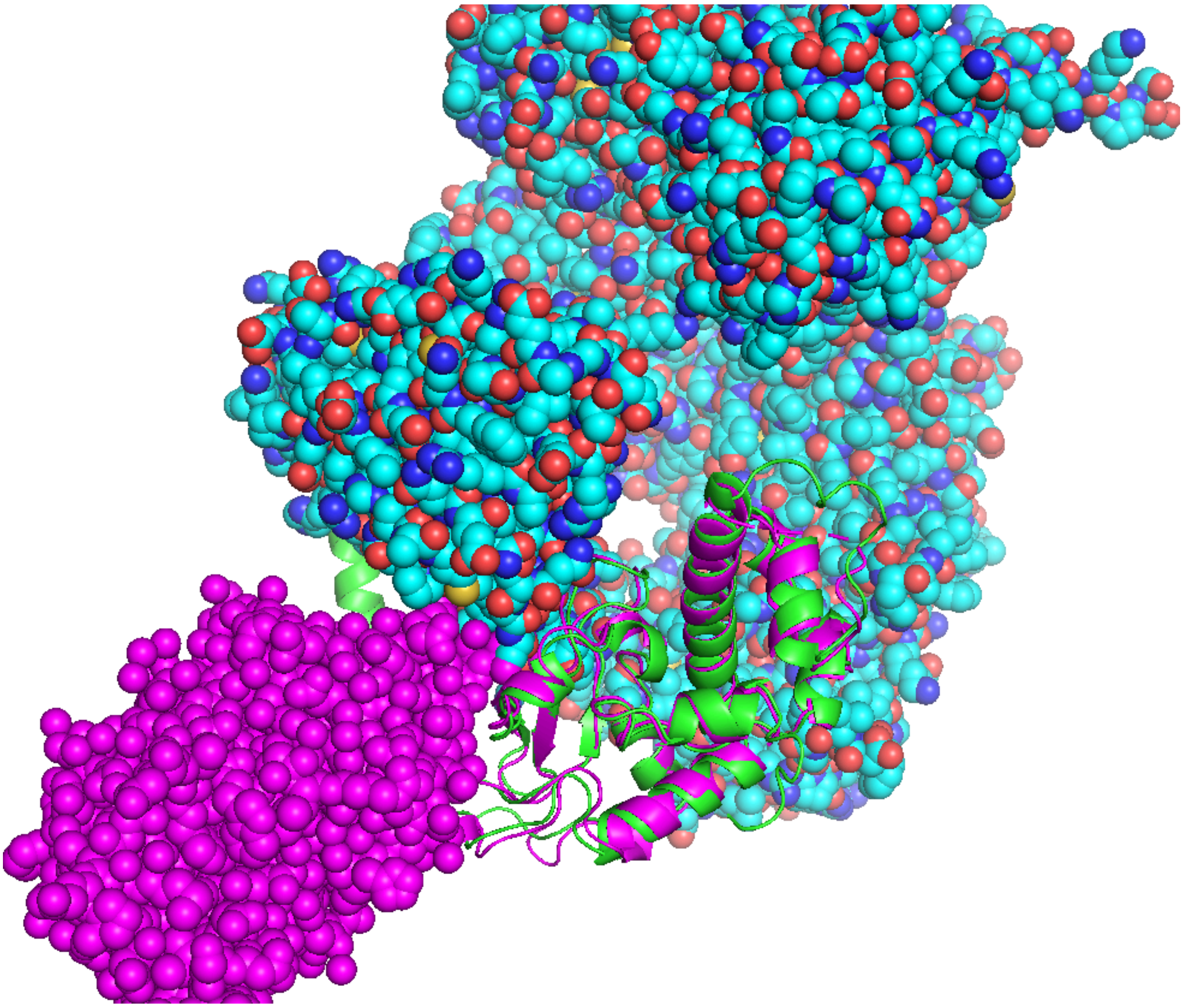

**Here is the predicted PDB file:**
https://gitlab.com/patrickbryant1/hpopt/-/blob/main/data/HPIDB/PDB/hc_preds/no_clashes/Y_pestis/P67870-A0A384KST0.pdb **and the dimer from PDB:**
https://www.rcsb.org/structure/1QF8

**As Protein kinase CK2 is a tetramer, any form of binding to an individual subunit likely inhibits the formation of the complex and, thereby, the activity.**

7. In Figure 4c, the authors report that approximately 25% of IGKC exists as a dimer, based on nMS experimental results. If so, was the IGKC dimer - IPD dimer - IGKC dimer complex observed? Since nMS data alone cannot distinguish between the IGKC dimer - IPD dimer (2:2) complex and the IGKC monomer - IPD dimer - IGKC monomer (1:2:1) complex, if such a structure is possible, the schematic representation should be revised accordingly.

**The reviewer is right that the possibility of a 2:2 complex cannot be excluded based on the MS data. However, we consider this a very unlikely scenario for two reasons:**

**1) The signal intensity for IGKC monomers is strongly reduced in the presence of IPD compared to the IGKC dimers (Figure 4c, middle and bottom panels). This suggests a**

**preferential interaction of the IGKC monomer with the IDP dimer that depletes free IGKC from the solution.**

**2) A 2:2 dimer poses a symmetry challenge. One possibility would be that one IPD subunit binds an IGKC dimer. In this case, the second subunit could also bind IGKC, however, we do not observe any signs of 2:4 or even 2:3 IPD-IGKC complexes. The other possibility is that the IGKC dimer binds at the interface of the IPD dimer, which would allow only a 2:2 complex. This would make the formation of a stable 2:1 IPD-IGKC complex unlikely, but we readily detect complexes with this stoichiometry (Figure 4 c).**

**For clarity, we have added a note to the text that a 2:2 complex of one IPD and one IGKC dimer cannot be excluded based on the MS data, but appears highly unlikely, especially given the fact that the predicted 1:2:1 structure shown in Figure 4b is in complete agreement with the MS results:**

*“ From these observations, we conclude that the most likely explanation is that the IPD dimer binds two IGKC monomers in a 1:2:1 architecture, in agreement with the AI model. We note that a 2:2 complex of one IPD and one IGKC dimer each cannot be excluded based on the MS data, but appears highly unlikely.”*

8. More generally, the stoichiometry alone cannot prove that the predicted structure is correct. For example, the authors may suggest a mutation (based on the predicted structure) that can change the stoichiometry and show that it actually works by using MS experiment. If additional data like this are not provided, the current logic is too weak.

**The reviewer suggests probing the exact nature of the dimer interface by predicting the effect of mutations and confirming their effect on dimer stability with native MS. Our previous work has shown that native MS can reliably reveal false positives in AI-based protein interaction predictions, it also showed that AI and MS differ significantly in their ability to accurately determine the effect of point mutations on protein interactions (https://pubmed.ncbi.nlm.nih.gov/35634779/). In fact, the only thing that can confirm that the predicted structure is correct is to determine the structure. As this is outside the scope of this work, we instead urge the reviewer to consider the data presented and the high likelihood of the predictions being accurate based on 1) the study of all known host-pathogen interactions in the PDB and the possibility to select these (Figures 1 and 2) the results from the nMS agreeing with the predictions and possible stoichimoteries. We do believe that the data presented confirms that the predictions are likely to be accurate.**

Minor comments
1. There are many grammatical errors. I strongly recommend the authors to review the manuscript thoroughly and carefully.

**Thanks for this comment. We have reviewed the grammar of the manuscript.**

2. The authors provide the hyperlinks of PDB and UniProt databases, but it should be avoided. Instead, I would suggest using PDB and UniProt IDs only. The authors may want to organize the hyperlinks into a separate table.

**This is a formatting issue that depends on the journal's requirements. If this is discouraged, we can, of course, change this, but we await the journal's instructions here as we have previously been advised to add the links after all PDB and Uniprot IDs.**

3. The manuscript presents the IPD-IGKC heterotetramer structure, but does not specify confidence metrics. If this structure was also predicted using FoldDock, the corresponding confidence scores should be reported.

**The heterotetramer shown in Figure 4 is based on the heterodimer structure predicted in this paper. To generate the heterotetramer, we applied simple homology modelling and combined two heterodimers into a heterotetramer by aligning each IPD protein in the heterodimer with a subunit of the native IPD dimer structure (1OJT) using the MatchMaker feature of ChimeraX-1.7.1. We have thereby not predicted the IPD dimer. We have made this clearer in the manuscript:**

To generate the heterotetramer (Figure 4b), two of the predicted IPD-IGKC heterodimers were combined into a heterotetramer by aligning each IPD protein in the heterodimer with a subunit of the native IPD dimer structure (PDB ID 1OJT). The model suggests that bound IGKC faces away from the dimer interface. If the prediction is correct, dimeric Francisella IPD should bind two IGKC monomers (Figure 4b).

4. The format of reference 2 is inconsistent with the other references.

**Thanks.**

**<u>Reviewer #2:</u>** Saluri et al. in their manuscript "AI-first structural identification of pathogenic protein targets," provide support for a subset of host-microbe interactions by performing an AlphaFold-based FoldDock method for structural prediction and then filtering high-confidence structures using author-defined criteria.

Overall claims:

The title and key claims of the paper are somewhat misleading, as the interactions that the authors investigated were already known prior to this analysis. The authors merely added

structural prediction onto the previous evidential data.

**We have changed the title to highlight that we identify novel interfaces: "AI-first structural identification of pathogenic protein target interfaces."**

The authors should highlight Dyer et al. 2010 in which the interaction between a bacterial dihydrolipoyl dehydrogenase and human IGKC was first identified.

The authors mention interface analysis, although this is missing from the results section, as the results are exclusively at the protein level.

**The results are indeed on the protein level. However, we generate novel interfaces which can inform the development of therapeutics which is the purpose of this study. We have changed the title to underline this better.**

It is unclear how the authors conclude that they have a 4-fold expansion in structures, as they provide 30 new structural predictions, but they claim earlier that there are 52 known complex structures.

**This is for human-pathogen interactions in the HPIDB from the 10 selected organisms. In the PDB there are structures for other organisms than what is captured in the HPIDB from the 10 selected ones, which is why these numbers differ. If we would consider all organisms in the HPIDB, these numbers would increase.**

Methods:

The authors justify using TM-score instead of DockQ by stating that DockQ does not perform well when native structures differ in length from predicted ones. However, they later used pDockQ in their analysis. Since pDockQ is derived from IF-plDDT and DockQ, wouldn't it also inherit the same limitations?

**We thank the reviewer for this point. It is true that pDockQ would inherit the same limitations, which is why we recalibrate on the TM-score. The pDockQ is merely the interface plDDT multiplied by the log(number of contacts) and is therefore neither dependent on the DockQ or TM-score, which is why it can easily be recalibrated to capture high-confidence predictions.**

The paper does not provide ROC analysis for TM-score thresholds below 0.9. TM-score ≥ 0.5 is already considered the same fold; I would like to see ROC curves for TM-score thresholds at 0.5 or 0.6.

**The reason for selecting such a high threshold is because we do realise that these are still predictions and, therefore, want to have very high confidence when calling a prediction accurate. We do not think it is useful to investigate lower confidence thresholds but leave this exercise to the reviewer or other interested parties as all data and scripts for analysis are available with instructions here, and the threshold for selection can be changed: https://gitlab.com/patrickbryant1/hpopt**

Results:

The statement in Figure 2d is confusing: “Figure 2d shows the number of HP-PPIs with known structure (n=10) and the number of high-quality predictions with an expected TM-score of 0.9 (above 70 plDDT and pDockQ 0.3, n=30). None of the interactions with known structures were accurately predicted.” Could the authors clarify whether “known structures” refer to monomers or complexes?

**Interactions always refer to complexes. No monomer modelling is assessed in this work. We do think that the “**the number of HP-PPIs with known structure” **is sufficiently detailed here as PPIs with known structure = protein-protein interactions with known structure.**

The HPIDB 3.0 database reports 55,505 unique protein interactions, yet the manuscript states 69,787 interactions in the first sentence of the “Human-pathogen PPIs from the HPIDB” section. Where does this discrepancy come from?

**This is due to updates in the HIPDB, which did not result in new versioning on their part. It currently contains the number we report. This information is also available at [https://gitlab.com/patrickbryant1/hpopt](https://gitlab.com/patrickbryant1/hpopt) .**

The authors claim to investigate a structural protein-protein interaction network, yet there is no actual network analysis presented in the study.

**The task of predicting all known HP-PPIs is the task of investigating the structural PPI network. We do, however, not claim to investigate this on the “network” level, as this is not the purpose of this study, but instead to investigate predicted interfaces and expected accuracies in these based on predicted confidence metrics.**

Minor:
Typo in Figure 4: IDP → IPD

**Thanks.**

## Reviewer #3: 
This paper reports the results of an effort to predict the 3D structure of host-pathogen protein-protein complexes. The predictions are performed using tools developed based on AlphaFold (AF) and AlphaFold-Multimer (AF-M), respectively trained on single protein chains and protein complexes from the PDB, previously developed by some of the authors. The predictions are performed on two datasets: a set of 111 (24) unique host-pathogen complexes with known 3D structure from the PDB and that of unique binary protein interactions of 9452 human proteins with ten different pathogens with unknown protein structures. The latter interactions, detected by various experimental techniques, were extracted from the HPIDB. Using various structure quality/reliability

measures, widely used by the community (with preference given to the TM score) the authors managed to correctly predict (recall) a small fraction of the known complexes and propose highly reliable 3D structures for a small fraction of the tested PPI set from HPIDB. The predicted structure of one of the host-pathogen PPIs from HPIDB was experimentally verified using native MS, and based on this experimental data the stoichiometry of the native complex was defined.

General comments.
The idea of applying AF and AF-M methods to obtain reliable information on the 3D structures of host-pathogen protein interactions makes great sense as it provides valuable information on the 3D structure details of the interacting partners that can be used to design vaccines or drugs likely to counter pathogen infections. The work is solid and represents means of advancing drug and vaccine design developments against pathogen infections.

**We humbly thank the reviewer for the nice description and understanding of our work.**

Minor comments: points demanding clarification.
1 Page 7: The fraction of reliably predicted structures (TM-score of 0.9) is rather low for the full set of 111 known structures and likely slightly lower for the subset of 24 structures that are more recent than those AF/AF-M were trained on (not explicitly shown but surmised from the average values). Why is this the case? What fraction of these two sets were determined by cryo-EM. What is the distribution of the resolution of these structures? Were cryo-EM structures les well predicted? Were lower resolution structures less well predicted?

**This is a very good question. We don't know why the fraction is low, but do not think that this is in any way related to resolution as all structures have resolution <5Å, which should be sufficient to resolve accurate interfaces. We do suppose this is related to the missing coevolution between the host and pathogen proteins making it more difficult for structure prediction tools to produce meaningful interactions. We have previously shown that the structure prediction of heterodimeric complexes is highly dependent on having accurate coevolutionary information, and the better this is, the more accurate the complex prediction will be (https://www.nature.com/articles/s41467-022-28865-w). Therefore, we expected this problem to be much more difficult as such information can't be obtained due to missing evolutionary links between host and pathogenic proteins. For all of these interactions, no paired MSAs can be generated: "**The resulting MSAs are block diagonalized as pairing should result in zero matches (the host and pathogen should not have common ancestral proteins). "

**Instead, the network has to rely on generating coevolutionary patterns throughout its internal representation based on learned principles. This is also the reason why we can't provide an analysis of what some interactions are more accurately predicted than others.**

2-Figure 2c: This Figure shows the distribution of the average plDDT for each chain in the predicted complexes from the HPIDB (n=8441) and the PDB (n=111), and the legend

mentions that “93 complexes out of 111 where both chains have over 70 plDDT on average for the PDB set (84%) and 3472 out of 7948 for the HPIDB set (44%)”. These numbers are much higher than the fraction of PPI with TM-Scores above 0.9 for PPIs from these two sources. This seems a bit surprising. It would therefore be helpful to provide a plot illustrating the relationship between the TM-Score and plDDT.

**This is the average TM-score of the proteins overall:** “Distribution of the average plDDT for each chain in the complexes”**. However, the pDockQ in Figure 1 relates to the interfaces where the plDDT will be much lower. This can be seen by the fact that very few of the PPIs have high pDockQ scores - consistent with Figure 1.**

3- Figure 2d plots the number of HP-PPIs with known structure (n=10) and with high-quality predictions (above 70 plDDT and pDockQ 0.3, n=30) for each pathogen. This information is provided here for the first time. What does ‘known structure’ mean in this context: The structure of the full complex or that of one of the partners (human or pathogen)?

**Known structure means that the complex is present in the PDB - not only the individual chains. We have added the word complex to underline this:** “Figure 2d shows the number of HP-PPIs with known complex structure (n=10)”

4-The question raised above is part a wider imperfection of this otherwise nice manuscript, which is, a lack of a master Table, or 2 such Tables (in the Suppl ), which list the for each PPI dataset (PDB and HPIDB,) all the relevant details : resolution + experimental method (X-ray/cryo-EM; AF/MS/2-Hb/other);origin (host pathogen); # of total predictions; # of predictions with TM-scores≥0.9; pDockQ >0.3; and plDDT >70% for both chain and any other info (diff in sequence length and fraction of disorder when known). And for the PPI’s from HPIDB also provide information on availability experimental structure(s). This will make it much easier to build on this work for future studies.

**We thank the reviewer for this suggestion. We do not think the resolution is of importance here as <5Å should be sufficient to resolve interfaces. We have provided such a table for the 10 studied pathogens:**

**Supplementary Table 1.** The number of structures in the PDB and high-confidence predictions obtained here for human-pathogen protein-protein interactions divided by pathogen.

| **Pathogen** | **Structures in PDB** | **High confidence predictions** | **Total** |
|---|---|---|---|
| Bacillus anthracis | 2 | 8 | 10 |
| Epstein-Barr virus | 4 | 1 | 5 |
| Human papillomavirus type 16 | 3 | 5 | 8 |
| Influenza A virus | 1 | 0 | 1 |
| Francisella tularensis subsp. | 0 | 4 | 4 |

**We have expanded this to include the total number of HP-PPIs, the number of predictions, predictions with sc pIDDT>70 and expected TM-score >0.9 as suggested:**

**Supplementary Table 1.** The number of structures in the PDB, the number of HP-PPIs in the HPIDB, the number of successful predictions, the number of these where both proteins have an average pIDDT>70 (single chain, sc, pIDDT) and high-confidence predictions (sc pIDDT>70 and pDockQ>0.3) obtained here for human-pathogen protein-protein interactions divided by pathogen.

| Pathogen | Structures in PDB | Number of HP-PPIs in the HPIDB | Number of successful predictions | Number of predictions with sc pIDDT>70 | High confidence predictions (sc pIDDT>70, pDockQ>0.3) | Total |
|---|---|---|---|---|---|---|
| Bacillus anthracis | 2 | 2882 | 2471 | 1170 | 8 | 10 |
| Epstein-Barr virus | 4 | 244 | 238 | 37 | 1 | 5 |
| Human papillomavirus type 16 | 3 | 171 | 158 | 59 | 5 | 8 |
| Influenza A virus | 1 | 238 | 230 | 53 | 0 | 1 |
| Francisella | 0 | 1295 | 1146 | 518 | 4 | 4 |

| | | | | | | |
|---|---|---|---|---|---|---|
| tularensis subsp. tularensis | | | | | | |
| Hepatitis C virus genotype 1b | 0 | 442 | 389 | 91 | 0 | 0 |
| Yersinia pestis | 0 | 3856 | 3405 | 1586 | 25 | 25 |
| Dengue virus type 2 | 0 | 134 | 104 | 30 | 0 | 0 |
| Human T-cell leukemia virus 1 | 0 | 77 | 76 | 0 | 0 | 0 |
| *Coxiella burnetii* | 0 | 237 | 224 | 49 | 0 | 0 |
| Total | 10 | 9576 | 8441 | 3593 | 43 | 53 |

5- The experimental validation of the predicted 3D structure of the IPD-IGKC, complex by native MS is very encouraging. Are there plans to carry out more extensive validations?

**We thank the reviewer for this remark. We plan to extend this work in the future to design inhibitors to predicted pathogenic interfaces and to also (potentially) resolve these structurally to show the full potential of the work presented here. We do however not have the possibility to do this in the scope of this study.**
