# Supplementary material for: AI-first structural identification of pathogenic protein target interfaces
Source: PLoS Comput Biol. 2025 Jun 26;21(6):e1013168. doi: 10.1371/journal.pcbi.1013168 (PMC12225977; doi:10.1371/journal.pcbi.1013168)
Supplement: Attachment — Submitted filename: Comments.pdf [file pcbi.1013168.s003.pdf]

1. The authors clearly explained in Figure 2d and the accompanying text that high-quality complexes were selected based on both plDDT ≥ 70 and pDockQ ≥ 0.3. Since pDockQ incorporates interface plDDT in its calculation, it is evident that the confidence of the binding site was at least partially taken into account.
   However, I still have some concerns about whether the pDockQ score reliably reflects the quality of the predicted interface.
   According to the manuscript, pDockQ is defined as:

$$pDockQ = \frac{0.724}{1 + e^{-0.052(x-152.611)}} + 0.018$$

   where x is "the average interface plDDT multiplied with the logarithm of the number of interface contacts (CBs within 8 Å)."
   In this formulation, even if the average interface plDDT is relatively low, a large number of interface contacts could yield a high pDockQ score.
   To clarify this point and avoid potential misinterpretation, I suggest including the average interface plDDT values for each complex in the supplementary material. This would help readers more accurately assess the actual quality of the predicted interfaces.

2. In response to the concern about the lack of structural basis in the functional interpretation of predicted complexes, the authors noted the existence of diverse inhibitory mechanisms and provided a detailed structural analysis for the CSNK2B case.
   I found this clarification helpful; however, I would suggest that similar minimal structure–function annotations for the other predicted complexes—such as the proximity of the predicted binding site to known functional domains or active sites—would further strengthen the overall reliability and interpretability of the results.
